# Board of Health Reports

**Published:** 1880-07

**Authors:** 


### BOARD OF HEALTH REPORTS.

WHEN a few months ago the Board of Health of the city of Buffalo initiated the publication of weekly reports, THE JOURNAL was prompt to express its approval, and our physicians generally considered it a move in the right direction. The little sheets were at first carefully read and placed on file on the Doctors' libraries. It soon became apparent, however, that as issued, they were of absolutely no value to the practicing physician, and we doubt if many of them now escape an immediate descent to the waste basket without even the formality of a removal of the wrapper. To see so much labor and money expended without any adequate return to the city is painful, but as now conducted it might more appropriately be called a Report of the Undertakers of the City of Buffalo, than of the Board of Health.

We do not make these statements in any spirit of captious criticism. We feel that the present Health Physician deserves much credit for so far overcoming the inertia which prevails upon this subject, as to be able to make a beginning in the right direction. With professional support and co-operation it is possible even with the present constitution of the board, to effect some changes in the report which would make it of great value. It is of interest to the statistician to know that so many died of pneumonia, cancer, &c., but to be of real use to the physician and the community at large, the report must go further. We want to know the death rate for prevailing diseases, the localities where disease is prevalent, and, if possible, the causes which occasion the sickness and mortality. A great amount of useful information might be thus gathered, and our efforts to effect sanitary improvements would be greatly aided by the ability to show the people how disease and deaths occur in any locality in direct proportion to its violation of sanitary laws. In the larger cities of Europe such information as to the localization of diseases is obtained by the simple expedient of sending each week to every physician in the city a blank upon which he makes a return of every case of contagious disease treated by him during the week, with a statement of the locality, number of street, and age of patient. These blanks are distributed by the police, and by them collected and turned over to the Health Physician, who upon these data, and supplemented with reports of sanitary inspectors, makes out his weekly report.

Some such system might be adopted in this city. To fill out such blanks would take but a few minutes of a physician's time, and information of great value might be thus obtained. We would thus be able to study the causes of, and the spread of, disease. We would be able to more justly estimate the mortality and suffering for which our city fathers are largely responsible, by practically compelling a large proportion of our poorer fellow citizens to use the polluted water from our public wells, and the deleterious influences on the public health of that perennial abomination, the Hamburg canal, would be soon abated if our weekly reports show that typhoid, scarlet fever, diphtheria, &c., are prevalent along the borders in much greater frequency than in other parts of the city.

The criminal carelessness which permits the continuance within the city limits of slaughter-houses and other business which may be considered in a sanitary light as public nuisances; the filling up of vacant lots with garbage, filth and dead animals; the filthy condition of the streets and tenement houses: these and many other sins against the public health would be recognized, if our little pamphlet shows how from such centres disease emanates with mathematical certainty, and how every violation of sanitary laws is followed by a sure punishment. It is only by bringing out such practical facts that we can hope to educate the people in sanitary science.

